# Vietnam biobanking feasibility study: An overview of biobanking landscape, infrastructure, and capacity

**DOI:** 10.3934/publichealth.2026013

**Published:** 2026-02-09

**Authors:** Hanh Vu, Io Hong Cheong, Jasper Hoi Chun Luong, Daniel Simeon-Dubach, Duyen Le, Thi Hong Xuan Pham, Nguyen Trung Thanh, Nguen Quoc Dat

**Affiliations:** 1 Vinmec Healthcare System, Hanoi, Vietnam; 2 University of Medicine and Pharmacy, Vietnam National University, Hanoi, Vietnam; 3 Faculty of Engineering and Information Technology, The University of Technology, Sydney, Australia; 4 Hainan International Medical Center, Shanghai Jiao Tong University School of Medicine, Hainan, China; 5 State Key Laboratory of Oncogenes and Related Genes, Center for Single-Cell Omics, School of Public Health, Shanghai Jiao Tong University School of Medicine, Shanghai, China; 6 Smoke-free & Healthy Life Association of Macau, Macau SAR, China; 7 Medservice, biobank & biobanking consulting and services, Hoerndlirain 22, 6318 Walchwil, Switzerland; 8 Hanoi Medical University, Thanh Hoa Campus, Vietnam; 9 Thanh Hoa Oncology Hospital, Thanh Hoa, Vietnam; 10 Institution of Science and Technology, University of Debrecen, Debrecen, Hungary; 11 Vietnam National Lung Hospital, Hanoi, Vietnam; 12 Institute of Biomedical Data and Science Research (BIODAS), Ha Noi, Vietnam

**Keywords:** biobanking, infrastructure, feasibility study, Vietnam, sample collection, management systems, capacity, challenges

## Abstract

Within Southeast Asia, there are still some challenges in managing non-communicable diseases and public health. Vietnam's government has supported its biotech research and pharmaceutical capacity for many years, including creating research infrastructure such as biobanks. To better understand the situation of biobanks in Vietnam, a feasibility study adapted from previous biobank work in Southeast Asia was conducted to analyze the knowledge gap for biobanking in Vietnam. The questionnaire was redefined for the purpose of the Vietnamese research climate, with 66 items categorized into 8 sections. Specifically, the study analyzed the following information: 1) General Information, (2) Biobank Information, (3) Sample and Storage Management, (4) Biobank Infrastructure, (5) Operational Resources and Personnel, (6) Laboratory Information Management Systems (LIMS), (7) Quality Management Systems (QMS), and (8) Risk Management Systems. The results showed that most biobanks in Vietnam have good energy supplies and backup power. Internet access was more critical, and backup sources were rare. Human resource management and training programs were well established. The QMS and LIMS were widespread, thus promoting confidence in the quality of biobanking, while waste management was also in place. However, reliable and available dry ice was hard to find, and sensitive data/documents lacked confidentiality. Most biobanks have a contingency plan, but alert systems/breach procedures raised questions about the quality of the contingency plans. To our knowledge, this is the first feasibility study of the biobank infrastructure in Vietnam. Our results, based on a selected group of biobanks, suggest there are well-established; however, there are critical challenges that should be addressed in the near future. Ultimately, the findings in this study should provide a foundation for future policy work, informed strategic planning, and resource allocation for the sustainable development and operation of biobanks in Vietnam. Additionaly, another survey with a broader group of biobanks should be planned.

## 1. Introduction

Over the past few decades, biotechnology research in Vietnam has seen considerable advancements, thereby prioritizing on agricultural applications aimed at improving crop yields and food security [1]. Over time, this focus was expanded to include medical biotechnology, with a rising emphasis on vaccine development [2],[3] and pharmaceuticals [4], as well as environmental biotechnology for sustainable practices [5]. To catalyze this growth, the Vietnamese government implemented various national strategies designed to enhance the research capabilities and foster innovation, supported by regulatory frameworks that govern the safe application of biotechnological advancements.

Strategically, the government planned to increase its pharmaceutical capacity through targeted investments and funding. These initiatives seek to fulfill 80% of the domestic demand and capture 70% of market value by 2030, with the ultimate goal for the industry to contribute $20 billion USD to the national GDP by 2045 [6],[7]. While foundational infrastructure such as biobanks has begun to emerge, establishing such sophisticated systems remain a challenge within the country. Sustained growth and the full realization of Vietnam's biotechnological potential depends on continued investments in research, education, and infrastructure.

Furthermore, critical infrastructure is essential to advance health service and achieve universal health coverage. This need is underscored by projections which indicate an increasing cancer burden in Hanoi and Ho Chi Minh City, driven by population growth and an aging demographic [8]. Although current models indicate a slow trajectory towards achieving the World Health Organization's Sustainable Development Goals by 2030, largely due to challenges in management at both a national and sub-national level, recent government support has accelerated progress [9],[10]. Despite these efforts, literature on biobanking infrastructure and research remains limited and inaccessible. Existing studies generally concentrate on niche collections such as human milk and cord blood [11]–[14].

Beyond Vietnam, the demand for biobanks is rising across the Association of Southeast Asian Nations (ASEAN) region, particularly highlighted by the recent ASEAN Biobank Feasibility Study [15]. This study underscored a strong demand to develop synchronized regional biobanks across different countries to enhance public health emergency responses. During this ASEAN study, a structured questionnaire tool was developed and applied using the Philippines as a case study. The survey instrument enabled a systematic evaluation of institutional readiness and technical gaps, and demonstrated how biobanks can refine health decision-making and foster research collaboration within the broader region. Similarly, research in the Middle East revealed resource requirements and operational limitations that influence the operations and sustainability of regional biobanking facilities [16]. Likewise, a survey of biobank development in China emphasized that human capital and professional development of staff and experts as invaluable resources for institutional success [17].

Advancing biotechnology in Vietnam requires a multifaceted approach that promotes national and international cooperation, adheres to regulatory, ethical and legal requirements, and encourages technological advancements to establish a sustainably and globally competitive research ecosystem. Key priorities include supporting public health and research initiatives and improving data management and augmenting funding opportunities. Centralization and standardizing the protocols for collecting, storing, and managing biological samples and data across individual facilities are essential to establish a national infrastructure for individual biobanks. This coordination will significantly enhance the efficiency of medical research, especially for biobanks with specific purposes such as cancer research [18], pathogenic microorganisms [19], infectious diseases [15],[20], and rare diseases [21]. Integrating these biobanks will facilitate more efficient access to samples and data, thus enabling more comprehensive and coordinated research efforts [22],[23]. A centralized biobanking model consolidates biospecimen storage and data management into primary hubs to ensure standardized quality control, while also leading to downstream adherence by associated biobanks and stakeholders. As such, they can lower overarching expenditures and expertise issues to maintain high quality samples [23]. However, logistical challenges, such as requirements for a highly efficient sample transport network, must be addressed to ensure the sustainability of such systems in Vietnam.

Distributing the benefits of such research across the population fosters a more equitable healthcare system. By ensuring that biological collections represent diverse demographics or specific demographics, the resulting medical treatments can be fairly distributed to reduce health disparities. This inclusive approach not only improves national healthcare outcomes, but also ensures that healthcare delivery is aligned with legal and ethical standards regarding an equal access to medical advancements. Furthermore, a standardized system facilitates the secure management of sensitive data. Ultimately, these factors can contribute to improved healthcare outcomes [24]–[26].

Overall, through this study, we seek to identify the foundational building blocks required to support this transition toward a national biobanking infrastructure. A feasibility study in Vietnam's biobanking scene would be invaluable to complement the knowledge gap on biobanking in the region alongside concurrent and ongoing efforts to improve best practices and regulatory efforts within the Southeast Asia and the Indo-Pacific Rim region [27],[28], taking the diverse cultural, linguistic, and legal contexts into account. Furthermore, regional studies are vital to increase public awareness and trust. This transparency is especially crucial when public resources are required to support public health infrastructure in resources limited settings like such Vietnam [29].

## 2. Materials and methods

### 2.1. Study design and setting

This cross-sectional survey was designed to assess the feasibility, infrastructure, and governance of biobanking in Vietnam. The study was developed based on previous biobanking feasibility assessments conducted by and during the ASEAN Biobank Feasibility Study [27], Qatar Biobank [30], and other comparable studies [31]–[35]. Such information has been unavailable regarding biobanking setup and infrastructure in Southeast Asia, specifically Vietnam. Biweekly virtual meetings were held by a panel of multidisciplinary advisors from Vietnam, China, and Switzerland, including clinical researchers, biobank managers, and governance specialists. These sessions were utilized to review available references and literature, identify operational needs, and collaboratively develop a biobank feasibility survey tailored to the Vietnamese context.

### 2.2. Instrument

The survey questionnaire was developed through several stages. First, the research team identified key domains of interest via a comprehensive review of relevant literature and previously validated biobanking questionnaires. Conceptual constructs related to biobank infrastructure, operations, and governance were mapped to develop an initial pool of items.

The preliminary questionnaire contained 66 items categorized into 8 sections: (1) General Information, (2) Biobank Information, (3) Sample and Storage Management, (4) Biobank Infrastructure, (5) Operational Resources and Personnel, (6) LIMS, (7) QMS, and (8) Risk Management Systems. The surveyed items have been summarized in Supplementary Table 1.

To check and ensure the clarity and feasibility, the draft was subsequently refined through iterative review sessions with healthcare professionals. Reviewers provided feedback on item clarity, terminology, and cultural or contextual appropriateness. Finally, the Vietnamese version of the questionnaire included minimal demographic information to preserve the respondent's anonymity.

### 2.3. Survey distribution and data collection

The survey was electronically distributed to professionals among the healthcare and scientific communities across Vietnam using a snowball sampling approach.

Distribution occurred from 06/01/2025 to 17/03/2025 via multiple digital platforms (e.g., institutional mailing lists, professional forums, and social media networks). The completion and submission of the online questionnaire constituted informed consent to participate in the study.

### 2.4. Data analysis

The information was collected by Google Forms and analyzed through Microsoft Excel. Quantitative variables were described as means (± standard deviation). The categorical variables were described as frequencies and percentages. The figures were graphed through ggplot2 and the tidyverse package of R Studio.

A particular emphasis was placed on aligning regulatory and governance structures, as well as addressing potential healthcare data integration requirements.

## 3. Results

The survey reached over 100 professionals, and 27 complete questionnaires were received and included in the analysis.

### 3.1. General information

The participants were asked to evaluate the necessity of high-quality biological samples for standardized biobanking using a five-point Likert scale as a multiple-choice format. The outcome was overwhelmingly positive, with 66.7% of the respondents rating the requirement as “Very positive” and 25.9% as “Positive”. The minimum response recorded for this item was “Neutral”, which accounted for 7.4% of the respondents, and no negative views were recorded on the quality requisite of samples for biobanks.

Supplementary Figure 1 demonstrates a broad agreement on the importance of all operational aspects to the establishment and sustainability of biobanks. Safety issues ranked the highest, with 92.6% of the respondents deeming them “Very important”, thus reaching a total of 100.0% when including those who said “Important”. Four additional components—appropriate documentation and standard operating procedures, collaboration between medical staff, researchers, and biobank personnel, infrastructure, and sufficient resources for operations—were identically rated as “Very important” by 81.5% of the respondents. Closely following were factors focused on personnel and security: properly trained staff was rated “Very important” by 77.8% of the respondents and security issues by 74.1% of the respondents. Finally, elements related to institutional management and external relations also showed significant importance. Financial sustainability was “Very important” to 70.4% of the respondents, while both general governance and community awareness were rated “Very important” by 59.3% of the respondents. Notably, across all operational aspects, the “Slightly important” and “Not important” categories were not utilized by any of the respondents, indicating that the minimum response for all items were neutral and higher.

### 3.2. Biobank information

The distribution of biobank types among the surveyed institutions showed that clinical, hospital-based biobanks account for the majority (50.0%) (Supplementary Figure 2). Academic biobanks affiliated with universities followed at 23.1%, while state-owned entities represented 15.4%. Commercial enterprises made up 7.7%, and disease-focused biobanks were the least common at 3.9%. The surveyed biobanks showed a mixed workforce structure, with both full-time (≥40 hours) and part-time (≤40 hours) staff present in biobanks (Supplementary Figure 3). 71.4% of the institutions surveyed employed full-time staff, with an average number of 8.8 full-time staff, a minimum of 2, and a maximum of 30.

Part-time staff are utilized by 51.8% of the biobanks, averaging 5.8 part time staff, ranging from 1 to 17.

### 3.3. Sample and storage management

Supplementary Figure 3 presents the distribution of specimen types across different biobank frameworks. Human biological specimens constituted the predominant component of the storage profile, as indicated by 85.2% of the biobanks. Microbial specimens rank as the second most prevalent (40.7%) and exhibit a more equitable distribution among clinical, academic, and state-owned biobanks. However, clinical biobanks represented the largest share of this category, equal to the combined contributions of academic, state-owned, and commercial entities. Animal specimens (18.5%) were preserved within academic and state-owned biobanks, as well as commercial entities. Fungal and plant specimens were underrepresented, each accounting for only 3.7% of the total storage profile, with their preservation largely confined to state-owned entities. It is noteworthy that commercial entities (7.7%) and disease-oriented biobanks (3.9%) only insignificantly contribute across all categories of samples.

Regarding specific human biospecimens, where all but three biobanks stored human samples, tissue samples were stored by 55.6% of biobanks, while plasma and serum were each preserved by 40.7% of all surveyed facilities. These frequencies of institutional storage are distinct from the total content percentages shown in Supplementary Figure 3.

### 3.4. Biobank infrastructure

Table 1 highlights a comprehensive overview of the critical infrastructure metrics of biobanks, thus revealing a generally favorable state of operational readiness alongside several significant deficiencies. 96.3% of biobanks indicated reliable access to electricity and internet services, with 81.5% possessed a backup power supply. Nevertheless, only 66.7% of biobanks reported having adequate space for prospective expansion, and a mere 18.5% maintained a backup internet connection. In terms of service reliability, 40.7% of biobanks affirmed the provision of an uninterrupted power supply, while only 14.8% indicated the availability of uninterrupted internet connectivity. Although essential utilities such as water were consistently accessible (92.6%), the absence of redundancy in internet systems, coupled with limited spatial capacity, may impede long-term scalability and resilience.

Moreover, in terms of operational equipment and supplies, biosafety cabinets and cryogenic freezers (−20 °C and −80 °C, respectively) were widely used across most sample types: they were used by over 87% of biobanks for BSL-2 laboratories and over 73.9% for freezers (−20 °C and −80 °C) (Supplementary Figure 4B). Freezers (−20 °C and −80 °C) were present in all facilities that stored animal, fungal and plant samples and in most microbial sample biobanks (90.9%), while about 73.9% of biobanks that stored human samples reported having such equipment.

The use of liquid nitrogen storage systems was also evaluated across the different biobanks: these systems were predominantly used by biobanks that managed animal samples, and reached a usage rate of 60% when such samples were stored. In contrast, a more moderate usage of liquid nitrogen storage systems was found for facilities with human specimens (34.8%), rare for microbial samples (18.2%), and absent for fungal and plant samples (Supplementary Figure 4A). Sophisticated apparatuses such as automated sample processing systems and DNA/RNA extraction technologies were thoroughly represented for fungal and plant specimens (100%), yet were underutilized for human (40.9% and 27.3%, respectively) and microbial samples (22.2% and 66.7%, respectively) (Supplementary Figure 4B). Microtomes and automated storage systems were the least used overall, with no utilization reported for fungal and plant specimens.

Humidity monitoring exhibited a comparable trend, with 59.3% relying on manual systems and 25.9% adopting automated systems. In contrast, parameters associated with gaseous components were significantly and less frequently monitored: 59.3% of biobanks did not engage in monitoring ambient O₂, and 70.4% abstained from monitoring the CO₂ concentrations. Only 4.0% of biobanks indicated the presence of automated systems to monitor the liquid nitrogen levels, and only 11.1% possessed automated systems to survey the ambient O₂ levels.

**Table 1.** Status of operational infrastructure for biobanks in Vietnam.

Characteristic	n = 27	%
Biobank has sufficient space to store samples and accommodate the future growth of collections		
Yes	18	66.7
No	9	33.3
Biobank has a stable power supply		
Yes	26	96.3
No	1	3.7
Frequency of reliable power supply		
Never interrupted	11	40.7
Rarely interrupted	11	40.7
Occasionally interrupted	4	14.8
No stable power supply	1	3.7
Biobank has a backup power supply		
Yes	22	81.5
No	4	14.8
In progress	1	3.7
Biobank has a stable internet connection		
Yes	26	96.3
No	1	3.7
Frequency of stable internet connection		
Never interrupted	4	14.8
Rarely interrupted	17	63.0
Occasionally interrupted	5	18.5
No stable internet connection	1	3.7
Biobank has a backup internet connection		
Yes	5	18.5
No	20	74.1
In progress	2	7.4
Biobank has a consistent water supply		
Yes	25	92.6
No	2	7.4

### 3.5. Operational resources and personnel

Table 2 illustrates that the majority of surveyed biobanks reported having established staff training, monitoring, and compliance procedures. Specifically, 77.8% of the respondents indicated the presence of training programs to ensure staff competence, while 70.4% reported periodic monitoring systems for staff performance and compliance. Similarly, documentation which verified that employees read and understood relevant SOPs was available in 66.7% of biobanks. In contrast to these findings, fewer institutions had formal recruitment policies and processes, as these were reported by only 40.7% of the facilities. Nearly an equal number of institutions were still developing these policies or lacked them altogether.

During biobanking operations, core laboratory and safety items were provided and available in most facilities: lab consumables were supplied by the highest proportion of biobanks at 88.0%, closely followed by disposable personal protective equipment at 84.0%. Additionally, dry Ice, which is essential for transport and short-term cold storage, was commonly provided by 74.1% of biobanks. Conversely, consumables linked to specific sample processing and long-term storage methods were less frequently supplied. The provision of Formalin and Liquid nitrogen was notably lower, at 44.4% and 37.0%, respectively. Finally, FFPE supplies are provided by the smallest fraction of biobanks, only 25.9%.

Followed by waste management methods, surveyed biobanks utilized chemical disinfection (81.5%) and on-site autoclave sterilization (77.8%) as the predominant waste management methods. Additionally, off-site autoclave services were used by 70.4% of biobanks. External waste handling was less frequent, including off-site incineration (33.3%) and third-party disposal (14.8%).

**Table 2.** Biobank operational resources, quality management and risk management (N = 27).

Characteristic	Yes		No		In development	
	n	%	n	%	n	%
Biobank operational resources						
Biobank has recruitment policies and procedures	11	40.7	7	25.9	9	33.3
Biobank developed training programs to ensure staff have the competency to perform their tasks	21	77.8	3	11.1	3	11.1
Biobank periodically supervise staff to ensure compliance in their work	19	70.4	3	11.1	5	18.5
Biobank developed documentation to confirm staff have read and understood all SOPs related to their tasks	18	66.7	4	14.8	5	18.5
Biobank's quality management system maintains records of the following activities						
Sample processing procedures	25	92.6	2	7.4	0	0
Laboratory procedures	26	96.3	1	3.7	0	0
Sample and related data distribution	20	74.1	2	7.4	5	18.5
Sample transportation and receipt	25	92.6	2	7.4	0	0
QA and QC for equipment and reagents	16	59.3	6	22.2	5	18.5
Equipment quality, maintenance, repair, and calibration	22	81.5	1	3.7	4	14.8
Safety programs	22	81.5	1	3.7	4	14.8
Medical and hazardous waste management	23	85.2	3	11.1	1	3.7
General emergency plans	18	66.7	2	7.4	7	25.9
Data collection	15	55.6	4	14.8	8	29.6
Participant consent forms	20	74.1	4	14.8	3	11.1
Personnel proficiency assessment plans	16	59.3	5	18.5	6	22.2
Occupational health and safety regulations	20	74.1	2	7.4	5	18.5
Biobank's risk management system						
Biobank has an emergency generator installed, regularly maintained, and capable of running critical equipment for a minimum of 48 hours	20	74.1	6	22.2	1	3.7
Biobank's computer and other electronic systems protected by an Uninterruptible Power Supply (UPS) or emergency power source	20	74.1	6	22.2	1	3.7
Biobank has an emergency plan to handle all emergencies (fire, floods, earthquakes, etc.) appropriate to its geographical location	1	3.7	24	88.9	2	7.4
Biobank has reliable biological sample transportation facilities or commercial shipment systems for domestic and international shipments	12	44.4	11	40.7	4	14.8
Biobank has policies to ensure the security of sensitive data and documents	17	63	6	22.2	4	14.8
Biobank has security alarm systems or personnel to maintain safety	6	22.2	16	59.3	5	18.5
Biobank has regulations for handling cases of damage, theft, loss, unexpected use, or misuse of stored samples	11	40.7	8	29.6	8	29.6
Biobank keeps backups of valuable samples at satellite location	1	3.7	24	88.9	2	7.4

### 3.6. Laboratory information management systems

Data collected regarding LIMS being used for sample cataloging and tracking across surveyed biobanks indicated that such systems were utilized by 37% of biobanks, and 33.3% reported that their system was “In development”, while 29.6% of biobanks do not use such a system at any capacity (Table 3).

Furthermore, as shown in Table 3, most facilities prioritized immediate data protection, with 66.7% of those reporting performing daily (50.0%) or weekly (38.9%) backups to any LIMS information (n = 18). However, data security was compromised by a lack of off-site storage, as a large majority of biobanks (70.4%) reported no backup of LIMS data to a separate physical location; only 14.8% did, with another 14.8% reporting this as “In process”. Furthermore, dedicated LIMS management staff was not yet standard practice, with 51.9% of biobanks reporting no dedicated team, compared to 40.7% which did have one (Table 3).

### 3.7. Quality management systems

Less than half of biobanks reported having a system to ensure the implementation of the latest version of standard operating procedures (SOPs), while 29.6% indicated such a system was still in development, as shown in Supplementary Table 2. The majority (59.3%) had an established QMS that described QA and QC activities, with a similar proportion complying with regional regulatory guidelines and undergoing regular audits or certifications. Compliance with national and/or international guidelines was reported by most biobanks (74.1%), whereas only a small proportion (7.4%–22.2%) indicated non-compliance across these quality management aspects.

In addition, Table 2 shows that documentation was most frequently available for laboratory procedures (96.3%), sample processing (92.6%), and sample transportation and receipt (92.6%). Additionally, records were commonly maintained for safety programs (81.5%), medical and hazardous waste management (85.2%), and equipment quality, maintenance, repair, and calibration (81.5%). In contrast, fewer biobanks kept records related to QA and QC for equipment and reagents (59.3%), data collection (55.6%), and personnel proficiency assessment plans (59.3%). A smaller proportion indicated that these documentation systems were still under development, particularly for emergency plans (25.9%) and data collection (29.6%).

### 3.8. Risk management systems

Emergency generators capable of supplying power to critical equipment for at least 48 hours were commonly available and appeared to be regularly maintained across 74.1% of the biobanks (Table 2). Additionally, the majority of biobanks (74.1%) protected their computer systems and other electronic equipment using uninterruptible power supplies (UPS) or equivalent emergency power systems. However, a small number of biobanks (22.2%) lacked such protection, thus placing them at greater operational risk, with only a single biobank planning to develop such systems (3.7%). Furthermore, only a single biobank (3.7 %) currently maintains a backup collection location for valuable samples at alternative locations (Table 2).

Reliable domestic and international specimen transport services were reported by 40.7% of biobanks. 22.2% of the facilities lacked formal policies that govern the confidentiality of sensitive data and documents, while 14.8% are looking into implementing them. Additionally, while 66.7% of the institutions reported having general emergency plans through QMS records, only 3.7% reported having emergency plans specifically for natural causes such as fires, earthquakes, and others. Additionally, 59.3% of the surveyed facilities lacked alarm systems to detect security breaches against break-ins and thefts.

**Table 3.** Reported laboratory information management system and backup frequency.

Characteristic	n	%
Presence of LIMS at biobank (n = 27)		
Yes	10	37.0
No	8	29.6
In Development	9	33.3
Frequency of biobank LIMS backup (n = 18)		
Daily	9	50.0
Weekly	7	38.9
Monthly	1	5.6
Irregular	1	5.6
Biobank has a dedicated LIMS team (n = 27)		
Yes	11	40.7
No	14	51.9
In process	2	7.4
Biobank backs up LIMS data in a separate location (n = 27)		
Yes	4	14.8
No	19	70.4
In process	4	14.8

## 4. Discussion

This feasibility study provides the first systematic overview of the biobanking infrastructure in Vietnam, based on data collected from 27 biobanks. The study aimed to assess whether the current infrastructure meets the requirements to establish a coordinated national biobanking framework.

Our findings indicate that there is strong support to establish standardized, high-quality biobanks in Vietnam, with nearly all participants expressing positive views. This enthusiasm reflects growing recognition of biobanking as a vital element of biomedical and clinical research facilities. Specifically, safety, documentation, collaboration, and infrastructure were related as the most important factors for biobank development. The high emphasis on safety and SOPs highlights awareness of biosafety and quality assurance needs, while collaboration between researchers, clinicians, and biobank staff were recognized as key to an efficient operation. Financial sustainability and governance were somewhat lower in ranking but still highly valued, thus indicating that while operational and technical foundations are well understood, long-term institutional and policy frameworks may still require strengthening.

The relatively lower emphasis on community awareness may reflect the early developmental stage of public engagement in biobanking in Vietnam, thus underscoring the need for educational and outreach initiatives to enhance societal understanding and trust in biobank-related research. To address the low emphasis of community awareness amongst even biobanking institutions, a comprehensive educational plan could be implemented. Examples of specific programs that can foster public awareness can include public awareness campaigns aimed at informing about societal benefits of biospecimen donations for personalized medicine. Furthermore, educational workshops dedicated to hospitals and academic institutions to help inform potential donors about the ethical protection and the long-term impact of their contributions could greatly influence public perceptions. Moreover, the development of transparent digital platforms for the public to access information regarding sample utilization and research outcomes can encourage public engagement and understanding of their impact. By establishing active dialogues between biobankers and community stakeholders, such initiatives can bridge the gap between technical infrastructure and societal support for long-term operational sustainability.

The participants of the survey were a selected group of biobanks in Vietnam, where a majority of the biobanks operated within a healthcare environment or for academic research. This distribution indicates that biobanking activity in the surveyed sample is primarily driven by the healthcare and academic sectors, with limited involvement from specialized or private-sector biobanks. The low proportion of disease-focused and commercial biobanks suggests potential gaps in targeted research infrastructure and private investment, thus pointing to opportunities for diversification and partnership development in the biobanking landscape.

In terms of staffing, 74.1% of biobanks employed full-time staff (mean = 8.8 persons) and 51.8% engaged part-time personnel (mean = 5.8 persons), as referenced in Supplementary Figure 2. The mixed workforce structure aligns with broader discussions in the biobanking community about the centrality of well-trained personnel for long-term sustainability. As Chróścicka et al. emphasized, “the core of the biobank should be composed of highly trained personnel” supporting quality, operations, and coordination” [36]. Therefore, increasing full-time staffing could be critical to support the implementation of SOPs, equipment handling, and long-term sample management plans.

The findings indicate that most Vietnamese biobanks are primarily oriented toward biomedical and clinical research, with human specimens representing the majority of stored materials (85.2%). This trend aligns with the dominance of hospital- and university-based biobanks, where patient-derived samples are more accessible and directly relevant to clinical studies. Additionally, microbial specimens are collected but to a lesser extent, while environmental, fungal, and botanical materials are rarely represented. This limited scope highlights potential opportunities to expand biobanking efforts beyond clinical domains to support agricultural, ecological, or environmental health research. Tissue specimens emerged as the most commonly stored type (55.6%), followed by plasma and serum (40.7%), thus reflecting a strong focus on disease-specific and pathology-related research. However, molecular biospecimens such as DNA and RNA were rarely preserved, which suggests a limited molecular processing capacity across most facilities. These results are consistent with findings from China, where hospital biobanks similarly prioritized tissue and blood derivatives over nucleic acid materials. Additionally, comparable trends were observed in other low- and middle-income countries, where human biospecimens dominated biobank collections while animal and plant samples remained underrepresented. Together, these patterns underscore the need for national coordination in Vietnam to strengthen molecular infrastructure and encourage the diversification of sample types to better support interdisciplinary research and future biotechnological innovation.

Although essential utilities such as water are consistently accessible, the absence of redundancy in internet systems, coupled with limited spatial capacity, may impede long-term scalability and resilience. The low prevalence of automated storage systems was observed across the surveyed biobanks, with a total utilization of 12.0%. When analyzed by specimen type (Supplementary Figure 4B/0, 13.6% and 11.1% of biobanks which store human and microbial samples, respectively, utilize such automation. This count implies that while essential equipment for routine operations is widely accessible, investments in automation and advanced molecular technologies remain limited. Such constraints may constrain precision research and large-scale data management capabilities.

While fundamental equipment is consistently accessible, the availability of specialized and high-throughput technologies remains constrained for specific sample types, notably human and microbial biospecimens. The enhancement of automation and cryopreservation infrastructure in these domains could substantially elevate biobank efficacy and research productivity.

Regarding environmental monitoring, most biobanks maintain a routine oversight of temperature and humidity, thus reflecting adequate adherence to standard biobanking practices. Nevertheless, only a small proportion employ automated systems to monitor cryogenic conditions or gaseous parameters such as O₂ and CO₂. This imbalance highlights a reliance on manual processes and raises concerns about biospecimen stability under fluctuating storage conditions. Strengthening automated monitoring systems, especially for cryogenic fluids and gas concentrations, would significantly improve risk management and ensure consistent preservation standards across biobanks. Furthermore, such systems can also allow one to improve the security and safety of biobank personnel, as automated detection of internal gas imbalances or container failures could mitigate risks during the handling and maintenance of cryogenic storage equipment.

Most biobanks reported well-established human resource management practices either already enacted or being in development, including staff competency assurance through training programs, SOP documentation, and compliance reviews. The training and competence of the staff ensure an internal quality control with sampling handling and storage, and can also increase the capacity to provide further personal development of workers and staff, as well as the capacity and capabilities of the biobank for further upscaling or technological implementations. Additionally, it is encouraging for the biobanks to develop dedicated recruitment policies within their human resource management, as dedicated staffing models for optimal operations could be analyzed for efficient and sustainable workings. As specialized biobanking staff are largely limited in Vietnam, it can be crucial to target staff recruitment based on the needs of the biobank's operations, where specialized or general skills could be required from its workers alongside concernment of having dedicated staff working full-time hours, especially as on-job training could be required for such a novel field of biobanking in Vietnam to ensure the quality of operations.

Waste management practices across facilities were consistent with standard biosafety expectations, with most biobanks employing chemical disinfection and autoclave sterilization to ensure the effective inactivation and sterilization of biohazardous materials. While the alignment of these practices with environmentally sustainable “green biobanking” principles [37] that minimize environmental footprint of biobanking operation warrants future evaluation, the limited use of incineration likely reflects regulatory restrictions concerning emissions.

Additionally, it is imperative to direct particular attention to the management of samples, as a notable minority have articulated concerns regarding the reliability and availability of dry ice and or liquid nitrogen as previously discussed. Although overall consumables were regularly stocked for regular operations in most surveyed biobanking facilities, especially PPE regular lab consumables, this is mostly indicative of the consumables being sourced due to the majority of facilities being a part of another facility such as for healthcare or research, and the uncertainty of dry ice and liquid nitrogen service is largely indicative that most facilities do not have a convenient supply for sample storage and handling in case of emergencies, which is also largely indicative from the risk management results.

The widespread use of QMS and LIMS, as well as documented compliance with various standards and regulations, is encouraging, as it promotes confidence in the quality of biobanking. Specifically, 70% of biobanks have been developing or are in use of LIMS, mostly for core functionalities such as sample logistics, compliance, and equipment status. However, the specific data that these biobanks are tracking could be further developed and integrated within their biobank workflow to increase the output efficiency while minimizing error. Currently, there is a significant percentage of biobanks that do not have LIMS implemented. These results also show opportunities for digital management adoption, specifically with barcodes and instruments/devices with laboratory information system (LIS) outputs, but can be limited by the available resources and infrastructure. Furthermore, the lack of confidentiality of sensitive data and documents in one third of biobanks has the potential to present a significant problem, with specimen de-identification and confidential document access control not being tracked, thus raising serious issues with privacy control.

Although the vast majority of biobanks report having a contingency plan in place, further questions about the established alert systems and breach procedures raised questions about the quality of contingency plans. Moreover, best practices to implement automated daily LIMS backups can be crucial to limit risk and emergencies, which even 50% of the biobanks surveyed that have any sort of LIMS implemented do not have the capacity to record daily LIMS backups; this can be highly indicative by the lack of dedicated LIMS teams, as only 40.7% do. The lack of dedicated LIMS teams can be a result of the lack of human resources for such personnel, thus having to place the burden of performing such tracking tasks to regular personnel with other responsibilities. Together with the relatively limited implementation of emergency response plans for physical catastrophes, combined with inadequate security measures to address theft and unauthorized access at biobanking facilities, raises concerns regarding the resilience and protection of critical biological resources, as well as the safety of working personnel. This gap in backup safety infrastructure represents a significant vulnerability for biobanks in Vietnam and warrants urgent attention to ensure the long-term preservation and security of valuable biospecimen collections.

Finally, the current infrastructure provides a good basis to further develop biobanking in Vietnam, depending on its needs for sample processing and storage. Critical operational infrastructure, such as energy supply, was present, and a back-up power supply was in place in almost all biobanks, thus guaranteeing power production for a minimum of 48 hours. Internet access was more critical; however, backup sources were not common to support information systems when down. Otherwise, computers and other electronic equipment were protected by UPS in almost all biobanks.

## 5. Conclusions

To our knowledge, this study represents the first feasibility assessment of biobank infrastructure in Vietnam. Findings from the participating biobanks indicated that individual infrastructures were generally well established; however, several critical challenges require timely attention. Notably, the predominance of healthcare- and human sample-focused biobanks in the survey may reflect a selection bias, as participation depended on available institutions willing to respond. Additionally, biobanking remains a relatively new concept in Vietnam; some respondents required guidance during the survey process, and uncertainties in their responses may have affected the data accuracy. As national awareness, training, and outreach initiatives expand, these limitations are expected to diminish, thus leading to broader and more reliable assessments in future studies.

Biobanking plays a pivotal role in strengthening the research capacity and advancing public health objectives, particularly in developing countries. Enhancing public trust and engagement in such initiatives will be crucial to foster sustainable growth and integration into national research systems. The results of this study aimed to inform policymakers, research institutions, and other stakeholders in Vietnam about the current capacities and areas for improvement, especially as the value of biobanks can be reflected in global research. Future studies should further examine financial sustainability, operational efficiency, and technical capability in comparison with other resource-limited settings to better evaluate the effectiveness of the existing biobanking practices.

## Use of AI tools declaration

The authors declare they have not used Artificial Intelligence (AI) tools in the creation of this article.


