# Supplementary material for: Vietnam biobanking feasibility study: An overview of biobanking landscape, infrastructure, and capacity
Source: AIMS Public Health. 2026 Feb 9;13(1):222–39. doi: 10.3934/publichealth.2026013 (PMC13084478; doi:10.3934/publichealth.2026013)
Supplement: Supplementary file 1 [file publichealth-13-01-013-s001.pdf]

Public Health

AIMS Public Health, 13(1): 222–239.
DOI: 10.3934/publichealth.2026013
Received: 31 October 2025
Revised: 30 December 2025
Accepted: 13 January 2026
Published: 09 February 2026

https://www.aimspress.com/journal/aimsph

Research article

Vietnam biobanking feasibility study: An overview of biobanking landscape, infrastructure, and capacity

Hanh Vu[1,2,3,*], Io Hong Cheong[4,5,6], Jasper Hoi Chun Luong[6], Daniel Simeon-Dubach[7], Duyen Le[8,9], Thi Hong Xuan Pham[10], Nguyen Trung Thanh[11] and Nguen Quoc Dat[12]

1. Vinmec Healthcare System, Hanoi, Vietnam
2. University of Medicine and Pharmacy, Vietnam National University, Hanoi, Vietnam
3. Faculty of Engineering and Information Technology, The University of Technology, Sydney, Australia
4. Hainan International Medical Center, Shanghai Jiao Tong University School of Medicine, Hainan, China
5. State Key Laboratory of Oncogenes and Related Genes, Center for Single-Cell Omics, School of Public Health, Shanghai Jiao Tong University School of Medicine, Shanghai, China
6. Smoke-free & Healthy Life Association of Macau, Macau SAR, China
7. Medservice, biobank & biobanking consulting and services, Hoerndlirain 22, 6318 Walchwil, Switzerland
8. Hanoi Medical University, Thanh Hoa Campus, Vietnam
9. Thanh Hoa Oncology Hospital, Thanh Hoa, Vietnam
10. Institution of Science and Technology, University of Debrecen, Debrecen, Hungary
11. Vietnam National Lung Hospital, Hanoi, Vietnam
12. Institute of Biomedical Data and Science Research (BIODAS), Ha Noi, Vietnam

* **Correspondence:** Email: hanh.vu@uts.edu.au; Tel: +84826230103.

Supplementary

Table 1. Questionnaire domains and question formats (66 items).

Domain/Items	Number of Questions			
	Multiple Choice	All That Applies	Short Answers	Written Response
Survey Consent/General Information				
Email, Full Name, Institution, NDA Agreement, and Signature, Biobank name, address, contact info/person, biobank type.	1	0	9	0
Biobank Information				
Initial reaction to biobank creation; rating importance of: Resources, Infrastructure, Financial Sustainability, Trained Personnel, Community Awareness, Collaboration, Documentation/SOPs, Safety, Security, and Governance	11	0	0	2
Biobanking Operations				
Biobanking Type, Employee counts (full/part-time)	1	0	2	0
Sample and Storage Management				
Sample types, specific sample details, and storage methods	0	2	0	1
Biobank Infrastructure				
Storage space/growth, satellite facilities, power reliability, backup generators, internet stability, backup internet, water supply, and use of specialized equipment; Monitoring systems.	8	0	0	1
Operational Resource & Personnel				
Personnel recruitment policies; training programs; staff supervision; competency documentation; and access to operational consumables (LN2, Dry Ice, Formalin, FFPE supplies)	6	0	0	1
Laboratory Information Systems (LIMS)				
Customization for tracking, types of data managed (patient data, barcodes, tracking, etc.), backup frequency, dedicated LIMS team, and off-site data backup.	4	1	0	0
Quality Management Systems (QMS)				
Presence of QMS (QA/QC), SOP version control, regulatory audits, national/international guideline compliance, and record maintenance for 11 specific laboratory/safety activities.	5	0	0	2
Risk Management Systems				
Emergency generator capacity (48h), UPS protection for electronics, emergency plans (fire, flood, etc.), transportation facilities, data security policies, alarm systems, and regulations for theft or misuse.	7	0	0	0
Other	1	0	0	1

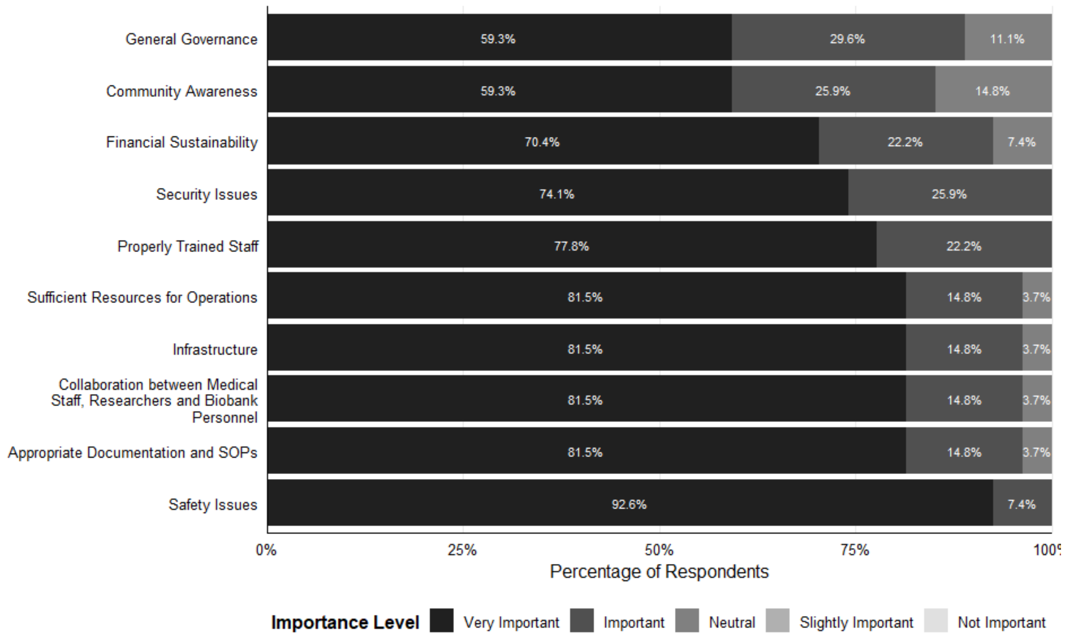

Figure 1. Perceived Importance of Biobank Operational Aspects by Vietnamese Biobankers. Distribution of importance levels assigned by respondents (N = 27) to ten distinct operational aspects of a biobank rated. Respondents evaluated each operational aspect using a five-point Likert scale, rating its importance from "Very Important" to "Not Important". Both English and Vietnamese version of survey are available upon request.

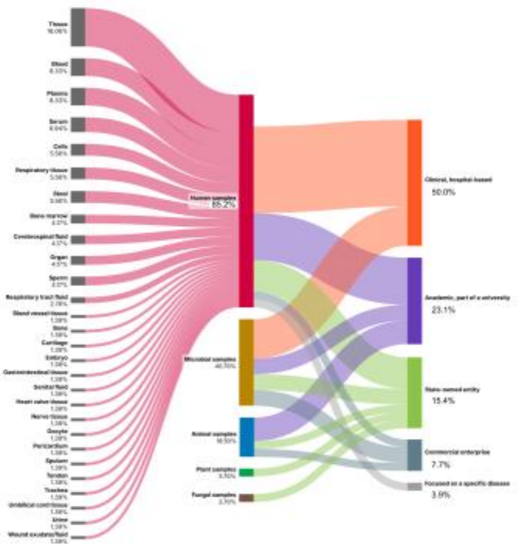

Figure 2. Sample Type Source and Biobank Affiliation. A Sankey diagram demonstrating the distribution and flow of samples from their initial material to the biobank affiliation. The left column lists 26 distinct primary sample materials collected by the participating biobanks. These materials are aggregated into five general source categories in the center column. The flow concludes at the right column, which identifies the five types of biobank affiliations. The width of the bands is directly proportional to the volume of the connection or flow.

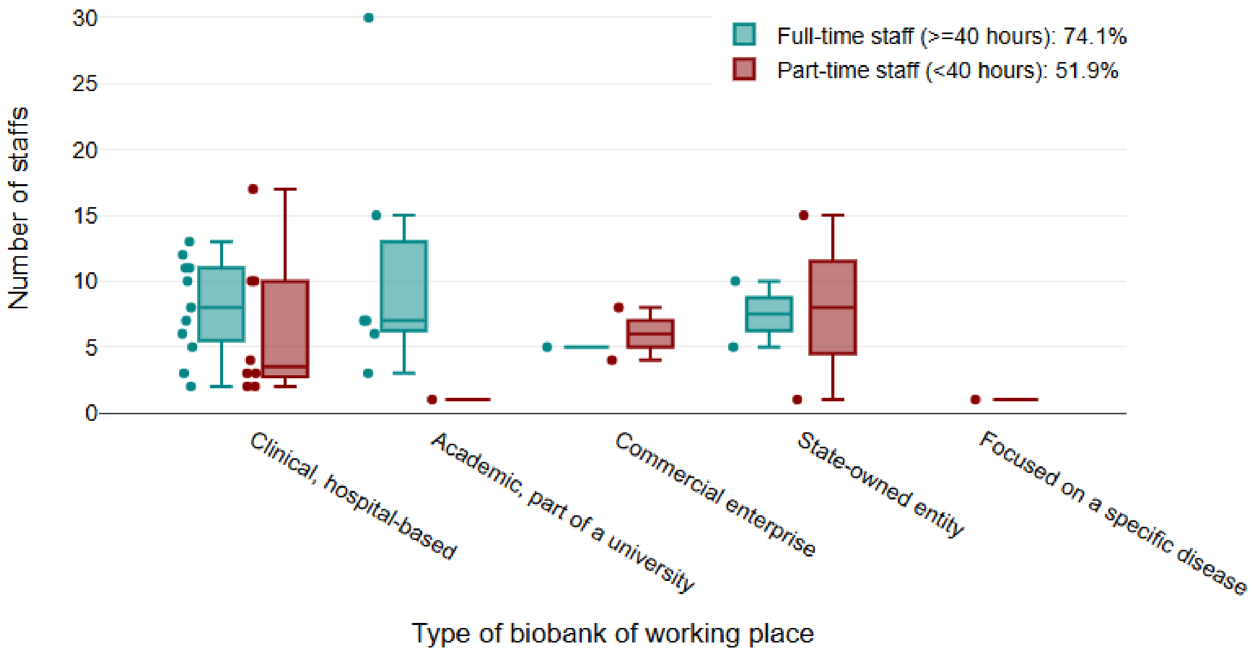

Figure 3. Distribution of Full-time and Part-time Staff Across Different Biobank Types. Boxplot of the number of staff members in various types of biobanks, categorized by employment status of full-time (40≥ hours) versus part-time (≤40 hours) across five biobank types.

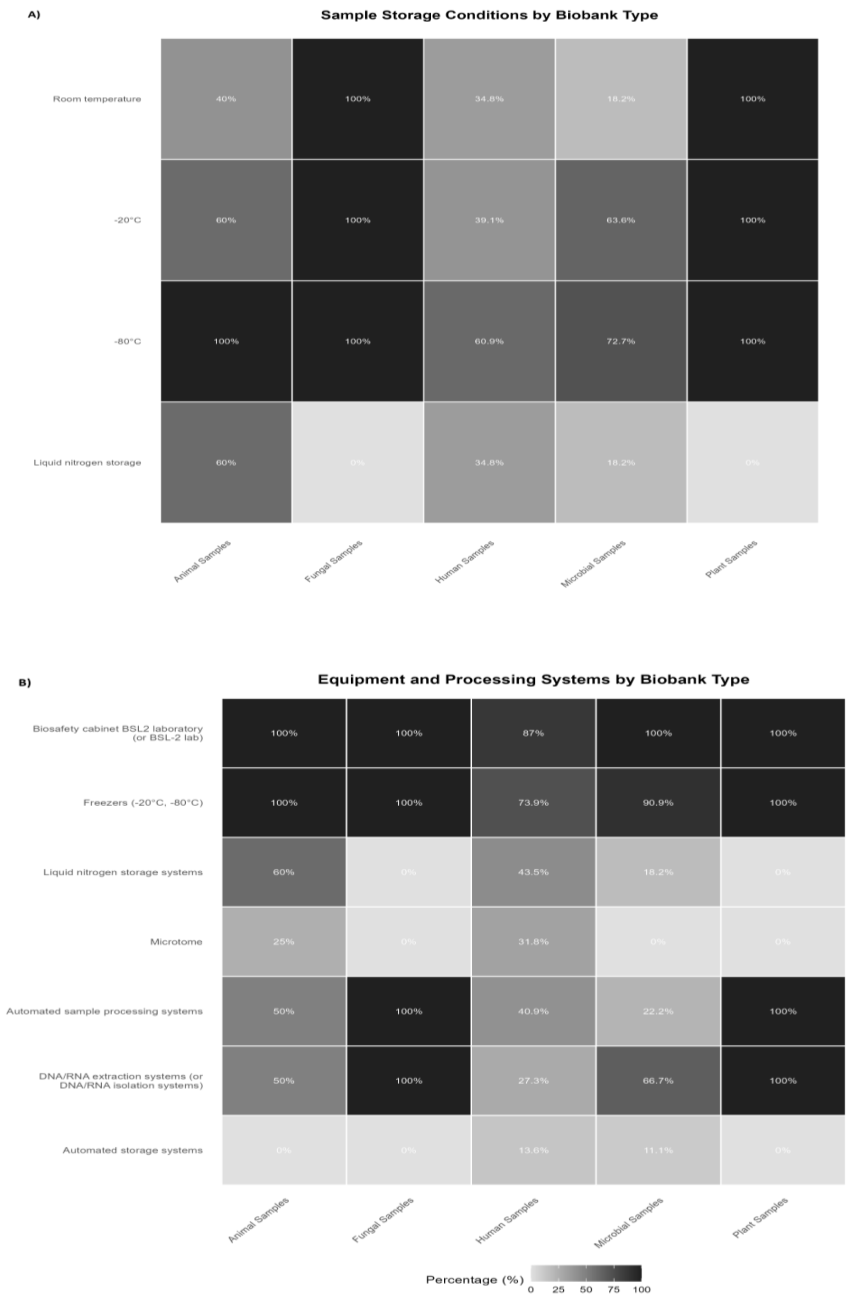

Figure 4. Sample Storage Conditions and Equipment by Sample Type. Heatmaps detailing the presence of specific storage conditions and processing equipment owned across biobanks categorized by the primary sample type they handle. The shade of gray in each cell is proportional to the percentage of biobanks that utilize the corresponding storage condition or equipment, as defined by the scale bar. 4A presents the distribution of four sample storage conditions across biobanks grouped by the five types of samples handled. The percentage of biobanks utilizing each storage condition is shown within the corresponding cell. 4B details the distribution of seven types of equipment and processing systems across the same five sample-type groups. The percentage of biobanks possessing each item of equipment is shown within the corresponding cell.

Table 2. Quality management system.

Characteristic	n	%
QMS for defining QA and QC activities		
Yes	16	59.3
No	3	11.1
In Development	8	29.6
Latest SOP versions were implemented		
Yes	13	48.1
No	6	22.2
In process	8	29.6
Regular regional audits were conducted		
Yes	16	59.3
No	5	18.5
In process	6	22.2
Multi-level guidelines were followed		
Yes	20	74.1
No	2	7.4
In process	5	18.5

AIMS Press

© 2026 the Author(s), licensee AIMS Press. This is an open access article distributed under the terms of the Creative Commons Attribution License (https://creativecommons.org/licenses/by/4.0)
